# Factors associated with actively working in the very long-term following acute coronary syndrome

**DOI:** 10.6061/clinics/2021/e2553

**Published:** 2021-01-18

**Authors:** Jose C. Nicolau, Remo H.M. Furtado, Talia F. Dalçóquio, Livia M. Lara, Marcela G. Juliasz, Aline G. Ferrari, Carlos A.K. Nakashima, Andre Franci, Cesar A.C. Pereira, Felipe G. Lima, Roberto R. Giraldez, Rocío Salsoso, Luciano M. Baracioli, Shaun Goodman

**Affiliations:** I Instituto do Coracao (InCor), Hospital das Clinicas HCFMUSP, Faculdade de Medicina, Universidade de Sao Paulo, Sao Paulo, SP, BR; II Hospital Israelita Albert Einstein, Sao Paulo, SP, BR; III Terrence Donnelly Heart Centre, St Michael’s Hospital, University of Toronto, Toronto, Canada

**Keywords:** Long-Term Post-Hospital Discharge, Return to Work, Acute Coronary Syndrome

## Abstract

**OBJECTIVES:**

Returning to work after an episode of acute coronary syndrome (ACS) is challenging for many patients, and has both personal and social impacts. There are limited data regarding the working status in the very long-term after ACS.

**METHODS:**

We retrospectively analyzed 1,632 patients who were working prior to hospitalization for ACS in a quaternary hospital and were followed-up for up to 17 years. Adjusted models were developed to analyze the variables independently associated with actively working at the last contact, and a prognostic predictive index for not working at follow-up was developed.

**RESULTS:**

The following variables were significantly and independently associated with actively working at the last contact: age>median (hazard-ratio [HR], 0.76, *p*<0.001); male sex (HR, 1.52, *p*<0.001); government health insurance (HR, 1.36, *p*<0.001); history of angina (HR, 0.69, *p*<0.001) or myocardial infarction (MI) (HR, 0.76, *p*=0.005); smoking (HR, 0.81, *p*=0.015); ST-elevation MI (HR, 0.81, *p*=0.021); anterior-wall MI (HR, 0.75, *p*=0.001); non-primary percutaneous coronary intervention (PCI) (HR, 0.77, *p*=0.002); fibrinolysis (HR, 0.61, *p*<0.001); cardiogenic shock (HR, 0.60, *p*=0.023); statin (HR, 3.01, *p*<0.001), beta-blocker (HR, 1.26, *p*=0.020), angiotensin-converting enzyme (ACE) inhibitor/angiotensin II receptor blocker (ARB) (HR, 1.37, *p*=0.001) at hospital discharge; and MI at follow-up (HR, 0.72, *p*=0.001). The probability of not working at the last contact ranged from 24.2% for patients with no variables, up to 80% for patients with six or more variables.

**CONCLUSIONS:**

In patients discharged after ACS, prior and in-hospital clinical variables, as well as the quality of care at discharge, have a great impact on the long-term probability of actively working.

## INTRODUCTION

Coronary heart disease (CHD) is a major public health burden worldwide. Besides being the leading cause of death globally, CHD is the greatest cause of disability-adjusted life years (1,2), with approximately 50% of patients suffering from myocardial infarction (MI) being of working age (3). Moreover, the estimated costs of care for CHD have risen dramatically in the last few years, especially as a result of the widespread use of new drugs, procedures, and imaging modalities for patient care (4). While these new treatments have changed the overall landscape in terms of short- and long-term survival (5), it is less established whether cardiac care during hospitalization translates into better patient-centered outcomes, such as the probability of being employed, especially in the long-term post-hospital discharge.

Loss of employment can have a profound impact on patient health and general welfare. Indeed, in a large US cohort, being unemployed for 1 year after a MI was associated with an increased risk of depression, lower quality of life, and higher hardship in affording essential post-MI medications (6). However, with the aging of the population, there is an increasing trend toward postponed retirement (7). In the US, the percentage of the labor force ≥55 years of age was 11.9% in 1996, with a projection to reach 24.8% by 2026 (8). This highlights the importance of healthcare systems being aware of factors that may influence the ability of post-MI patients to resume work and remain employed in the longer-term.

Factors associated with the probability of being employed after acute coronary syndrome (ACS) have been previously reported in cohorts from different countries. Factors such as depressed mood, left ventricular dysfunction, arrhythmias, or heart failure during hospitalization have been independently associated with a lower probability of returning to work (9-11). The association between delayed time to primary percutaneous coronary intervention (PCI) and employment status after MI has been controversial. While clinical factors such as heart failure (HF) and chronic kidney dysfunction have also been implicated, the role of baseline demographics such as age and sex is still disputed (12,13). Moreover, HF and diabetes have also been associated with a higher likelihood of loss of employment after 1 year (14).

All the aforementioned reports had no more than 5 years of follow-up, and none of them were performed in a developing country. Therefore, we sought to undertake a comprehensive analysis of how demographics, health insurance issues, clinical factors, and treatment delivered during hospitalization were associated with the longer-term probability of working after an episode of ACS.

## MATERIALS AND METHODS

### Study population and data collection

We retrospectively analyzed 2,816 patients with ACS who were included in a dedicated administrative databank from a quaternary care cardiology hospital between 1998 and 2014 and followed for up to 17 years. The variables included in the databank were collected on a routine basis for quality assurance (not necessarily for research purposes), and the requirement for consent was waived according to local regulations. For the purpose of the present publication, all data were retrieved anonymized from the databank.

**Supplemental Figure 1:**
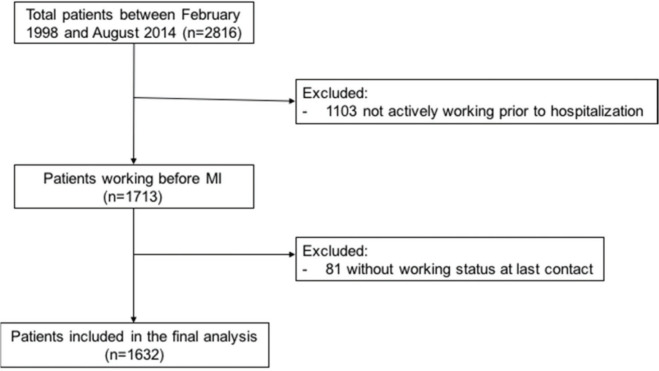
Flowchart of the study patients.

From the global population, we excluded 1,103 patients who were not actively working immediately prior to hospitalization, and 81 for whom the information about work status at the last contact was not retrieved; thus, the final analyzed cohort consisted of 1,632 patients (Supplemental Figure 1).

The majority of the patients were not followed-up in the institution; instead, a telephone contact was implemented yearly by undergraduate medical students who were specifically trained for the task and supervised by one of the authors (JCN). A standardized questionnaire containing information that included the incidence of MI after hospital discharge was administered to all patients and/or relatives.

### Statistical analyses

The incidences of the analyzed variables are reported as numbers (%). The probability of the patient to be working at the last contact was estimated by Kaplan-Meyer curve. Twenty-four variables that could potentially be associated with actively working (or not) at the last contact, such as the type of health insurance, age, previous MI, recanalization therapy, and proven medications at discharge, were selected and are described in Table 1. Univariate Cox regression was used to analyze the association between each variable and actively working at the last contact. Patients were analyzed by considering the last available information and were censored after death or last contact. In order to analyze the variables that were significantly and independently associated with actively working at the last contact, we included working status at the last contact as the dependent variable, and the others included in Table 1 as independent variables. The stepwise Cox regression test was applied for these analyses in three different models: In the first, only the baseline variables were included as independent variables, the second model included the baseline and in-hospital variables as independent variables, and finally, the third model included baseline, in-hospital, and discharge/after discharge variables as independent variables. Finally, we developed a prognostic index including the variables with a hazard-ratio (HR) <0.80 (inverse correlation) with active working at the last contact in Cox regression model 3 (age, previous angina and MI, anterior MI location, fibrinolytic utilization, in-hospital cardiogenic shock, and new MI during follow-up). The log-rank test was used to analyze the correlation with not working at the last contact, and the ROC curve was used to analyze the discrimination performance of the model.

## RESULTS

The final 1,632 patients analyzed in the present study were followed-up for a mean duration of 6.98±0.10 years (mean survival time: 9.3±SE 0.14 years). The median age of the population was 58 years (25^th^-75^th^ percentiles 51-66) and 77.4% were male. Table 1 shows the clinical characteristics of the study population; 29.1% had private health insurance, while the remaining 70.9% had only government health insurance. The prevalence of diabetes, hypertension, dyslipidemia, and current smoking was 25.8%, 69%, 55.7%, and 28%, respectively. Approximately 40% of the population had ST-elevation myocardial infarction (STEMI).

Table 2 shows the probability of working at the last contact according to the variables analyzed. Government health insurance, primary percutaneous coronary intervention (PCI), and statin/beta-blocker/angiotensin-converting enzyme (ACE) inhibitor/angiotensin II receptor blocker (ARB) prescription at hospital discharge were positively associated with long-term work. In contrast, a history of angina, STEMI, use of fibrinolytic, in-hospital coronary artery bypass grafting (CABG), and (re)MI after hospital discharge showed negative correlations with active working at the last contact.

Table 3 and Supplemental Tables 1 and 2 show the variables that were significantly and independently associated with actively working at the last contact in the three different models. From the baseline variables included in the three models, government health insurance, history of angina, prior MI, STEMI, and anterior-wall location MI showed a significant correlation in the three models developed. From the in-hospital variables analyzed in two of the three models, fibrinolysis and in-hospital CABG correlated significantly with active working at the last contact in both. Finally, in model 3, which included all the analyzed variables (Table 3), those that correlated positively with active working were as follows: government health insurance (hazard-ratio [HR], 1.36; 95% confidence interval [CI], 1.16-1.6; *p*<0.001), statin (HR, 3.01; 95% CI, 2.43-3.71; *p*<0.001)/beta-blocker (HR, 1.26; 95% CI, 1.04-1.53; *p*=0.020)/ACE inhibitor/ARB (HR, 1.37; 95% CI, 1.14-1.65; *p*=0.001) prescription at hospital discharge, and male sex (HR, 1.52; 95% CI, 1.24-1.85; *p*<0.001). Negative correlations were obtained for previous angina (HR, 0.69; 95% CI, 0.58-0.82; *p*<0.001) and MI (HR, 0.76; 95% CI, 0.63-0.92; *p*=0.005), smoking (HR, 0.81; 95% CI, 0.69-0.96; *p*=0.015), in-hospital non-primary PCI (HR, 0.77; 95% CI, 0.65-0.91; *p*=0.002), fibrinolysis (HR, 0.61; 95% CI, 0.47-0.78; *p*<0.001), cardiogenic shock (HR, 0.60; 95% CI, 0.38-0.93; *p*=0.023), MI after hospital discharge (HR, 0.72; 95% CI, 0.58-0.88; *p*=0.001), and age above the median (HR, 0.76; 95% CI, 0.54-0.89; *p*=0.001).

**Figure 1:**
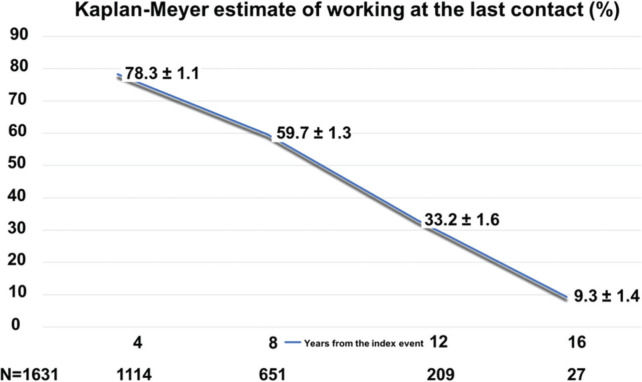
Kaplan-Meyer estimates for working over the course of time from the index event.

Figure 1 shows the Kaplan-Meier estimates for the patient to be working according to time from the index event. As expected, the probability decreased steadily, with 78.3% at 4 years, and 9.3% at 16 years.

**Figure 2:**
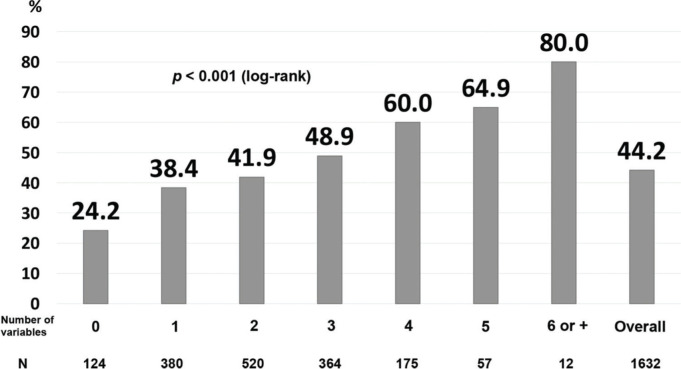
Prognostic index for not actively working at the last contact. The prognostic index included the following variables: history of angina, myocardial infarction, anterior-wall location on ECG, non-primary percutaneous coronary intervention, fibrinolysis, cardiogenic shock, myocardial infarction after hospital discharge, and age above the median (log-rank *p*<0.001).

Finally, we developed a prognostic index that included the variables that correlated significantly, independently, and negatively with actively working at the last contact (Table 3). The area under the ROC curve was 0.60. For the patients without any of the risk factors, the probability of not working at the last contact was only 24.2%, while the percentages increased proportionally with the number of risk factors: 48.9% for those with three risk factors, and 64.9% for those with five risk factors (Figure 2).

## DISCUSSION

In 1,632 post-ACS patients followed-up for a median of 7 years, and up to 17 years, we observed several key findings. First, the overall probability of not returning to work was less than 50%, a proportion that was comparable to other publications, but with a greater duration of follow-up post-ACS than previously reported (6,11-19). Second, clinical factors related to performance measurements from the health system (such as undergoing primary PCI or the prescription of statins or beta-blockers at discharge) were positively associated with active work during long-term follow-up. Third, there was a decrease in the percentage of patients actively working after the fourth year post-hospital discharge, and this percentage appeared to stabilize around 50% after the initial period. Fourth, a prognostic index for predicting working status after ACS was able to identify patients who were less likely to resume employment in the long-term, with an incremental higher risk of not working across the gradient of scores from 0 to 7. This information may be of relevance to healthcare systems, since those patients deemed to be at higher risk of not working in the long-term could be identified soon after discharge; this could allow for interventional programs focused on improving patients’ ability to return to and maintain active working to be implemented.

Factors associated with the probability of withdrawing from employment, retiring, or being dismissed after an episode of ACS were described. Among demographic factors, previous studies have consistently found an association between a lower probability of working after ACS and female sex (14,16). However, this is not unanimous, since this association was not observed in the study by Bhattacharyya et al., although the numerical trends persisted and the number of included patients was much lower than other prior publications (11). In a dedicated registry of young (*i.e.*, ≤55 years old) post-MI patients, Dreyer et al. found that the association between female sex and lower probability of working was mostly explained by confounders related to differences in demographic, occupational, and health characteristics between men and women (16). In our study, with a longer follow-up than that in previous reports, male sex was independently associated with a 1.5-fold higher probability of being actively working compared to female sex.

Among clinical factors, some prior reports have focused on markers of ACS severity and/or quality of care received at the index event. Nielsen et al. observed that a low ejection fraction (≤35%) measured by echocardiogram was associated with almost two-fold the risk of retirement at 4-year follow-up (10). Laut et al., in a Danish registry, found that a delay in reperfusion, defined as time from first contact to reperfusion therapy in STEMI >120 minutes, was independently associated with a lower probability of return to the labor market (12). Conversely, Isaaz et al., in a French registry, found no independent association between time of reperfusion or LV function and the likelihood of returning to work among patients with STEMI (13). Whether these discrepancies can be explained by different study designs, sample sizes, or socioeconomic characteristics of each country cannot be determined. In our data, not only clinical risk factors from the index event, but also markers of quality of care were significantly associated with better employment outcomes. The use of statins, beta-blockers, and ACEI/ARB at hospital discharge were positively associated with work status following ACS. Regarding statins, there was a 3-fold higher probability of being employed at a mean time of 7 years for patients who were prescribed statins at discharge. This is perhaps not surprising considering the clinical trial evidence of significant decreases in CV events in patients using statins, including events that may impact longer-term work ability, such as subsequent MI and stroke (20). In agreement with this, we also observed that having a new ACS event during follow-up had a negative association with working status, similar to the findings of Bhattacharyya et al. (11).

Taken together, these findings reinforce that performance measurements related to quality of care are strongly associated with working status after ACS. This underscores the need to improve adherence to guideline-directed medical therapies. In a prior publication from our group from the BRACE (Brazilian Acute Coronary Syndromes) registry, we observed that such performance measures in our country were less than ideal, and that achieving a high standard on these quality parameters was associated with better outcomes, including survival (21). Therefore, the same impact may exist regarding long-term employment status, and this issue may be addressed in future studies.

Besides clinical factors and quality of care, psychosocial factors may be related to working status after discharge for ACS. In a Japanese registry, Soejima et al. found that the presence of depressive symptoms during hospitalization and introverted personality were associated with a higher failure to return to work after MI. However, the study was limited to married men aged <66 years (9). In another registry from the UK, Bhattacharyya et al. observed that higher depression scores, measured by the Beck Depression Inventory, during index hospitalization were associated with a lower probability of returning to work after 12 months, independent of clinical covariates (11). These results were confirmed by Jonge et al. in a more contemporary registry from the Netherlands (19). Moreover, in a French registry, Isaaz et al. observed that unmarried status at the time of MI was associated with a lower probability of resuming work at a median of 42 months after MI (13). In one randomized controlled trial, Fors et al. demonstrated that a program of patient-centered care, focused on self-efficacy and active patient involvement in treatment decisions, resulted in a better quality of life but failed to increase the rates of returning to work at 6 months (22). In our study, we found that having government-funded health insurance, compared to private health insurance, was independently associated with higher rates of work retention. Considering that government-insured patients usually have a lower socioeconomic status than private-insured ones, this finding stresses the great inequality observed in our country, leading the lower strata of the population to continue to work even, in some cases, under unfavorable medical and environmental conditions. Therefore, the play of chance and unknown confounders could also be reasonable explanations for this association.

Our study has some strengths and novelties that should be highlighted. First, since the vast majority of evidence regarding work status after ACS comes from registries in developed countries, understanding the reality from a developing country may be important from a global healthcare perspective. Second, our follow-up time is longer than that of previous reports on the topic. Third, we developed a prognostic index to ascertain the probability of not working in the long-term, which could be an important tool to help clinicians decide how to manage socioeconomic needs from patients upon discharge after ACS.

We acknowledge that our study has several limitations. First, although our registry has a considerable size compared to previous reports, since we analyzed patients from a single center, it is not possible to determine whether our findings could be replicated in other parts of the country, or even in other countries. Second, we did not collect data regarding economic and psychological status during hospitalization besides health insurance. Third, we had no information regarding the types of jobs that were done by the patients, such as laborious work *versus* administrative, clerical occupations. Fourth, we only assessed the performance measures at discharge, and it is possible for them to have changed during follow-up. Fifth, although our score could identify an incremental risk across categories, it did not have an external validation cohort. Finally, the associations between statins, beta-blockers, and ARB/ACEI at discharge might be explained by residual confounders, since those treatment allocations were at the discretion of the attending physician.

## CONCLUSIONS

In patients discharged after ACS, variables related to the clinical severity of the acute episode and the quality of care delivered at discharge were associated with the likelihood of actively working in the long-term. These findings underscore the need to improve adherence to guideline-directed therapy as an important tool to potentially impact patient-centered outcomes.

## AUTHOR CONTRIBUTIONS

Nicolau JC and Furtado RHM were responsible for the conception and design of the research. Dalçóquio TF, Lara LM, Juliasz MG, Ferrari AG, Nakashima CAK, Franci A, Pereira CAC were responsible for the data acquisition. Salsoso R, Giraldez RR, Baracioli LM, Lima FG, Dalçóquio TF, Furtado RHM, Goodman S and Nicolau JC were responsible for the data analysis and interpretation, and critical revision of the manuscript for intellectual content.

## APPENDIX

## AUTHOR CONTRIBUTIONS

Montenegro LR contributed to the acquisition, analysis, interpretation of data, and drafting of the article. Lerario AM contributed to the interpretation of data and revising the article. Nishi MY contributed to the interpretation of data, drafting and revising the article. Jorge AA contributed to the analysis and interpretation of data. Mendonca BB contributed to the conception and design of the study, drafting and revising the article.

## Figures and Tables

**Table 1:** Clinical characteristics, treatments, and work outcomes of the population.

Variables (%)	Presence at baseline	Working at the last contact
Age > median	781 (47.9)	376 (48.1)
Male sex	1263 (77.4)	511 (40.5)
Government health insurance	1157 (70.9)	554 (47.9)
Previous angina	482 (29.5)	249 (51.7)
Previous PCI	306 (18.8)	160 (52.3)
Previous stroke	49 (3.0)	30 (61.2)
Previous CABG	219 (13.4)	120 (54.8)
History of diabetes	421 (25.8)	208 (49.4)
History of hypertension	1126 (69)	534 (47.4)
History of dyslipidemia	909 (55.7)	410 (45.1)
History of MI	455 (27.9)	252 (55.4)
History of HF	98 (6.0)	62 (63.3)
Smoking	490 (27.9)	199 (40.6)
STEMI	661 (40.6)	264 (39.9)
Anterior-wall MI	482 (29.6)	197 (40.9)
Primary PCI	358 (21.9)	217 (60.6)
Non-primary PCI	613 (37.6)	331 (54)
Fibrinolysis	184 (11.3)	106 (57.6)
In-hospital CABG	236 (14.5)	112 (47.5)
In-hospital cardiogenic shock	52 (3.9)	21 (40.4)
Statin at hospital discharge	1073 (80.2)	458 (42.7)
Beta-blocker at hospital discharge	1092 (81.6)	480 (44.0)
ACE inhibitor/ARB at hospital discharge	1000 (74.7)	439 (43.9)
MI at follow-up	303 (18.6)	155 (51.2)

Percentage (%) is the proportion of patients with an outcome. PCI: Percutaneous coronary intervention, CABG: Coronary artery bypass grafting, MI: Myocardial infarction, HF: Heart failure, STEMI: ST-elevation myocardial infarction, ACE: Angiotensin-converting enzyme, ARB: Angiotensin-receptor blocker.

**Table 2:** Probability of working at the last contact by Cox regression univariate analyses according to baseline, in-hospital, and after discharge variables.

Variables	Hazard-ratio (95% CI)	*p*-value
Age > median	0.90 (0.79-1.03)	0.137
Male sex	1.33 (1.12-1.58)	0.001
Government health insurance	1.21 (1.05-1.39)	0.007
Previous angina	0.72 (0.62-0.84)	<0.001
Previous PCI	0.98 (0.82-1.18)	0.862
Previous stroke	1.07 (0.68-1.68)	0.782
Previous CABG	0.88 (0.71-1.09)	0.240
History of diabetes	0.97 (0.83-1.13)	0.710
History of hypertension	0.94 (0.82-1.08)	0.395
History of dyslipidemia	1.06 (0.93-1.21)	0.392
History of MI	0.87 (0.74-1.01)	0.074
History of HF	1.06 (0.76-1.84)	0.724
Smoking	1.01 (0.88-1.16)	0.920
STEMI	0.89 (0.76-0.99)	0.038
Anterior-wall MI	0.89 (0.77-1.02)	0.104
Primary PCI	1.39 (1.19-1.63)	<0.001
Non-primary PCI	0.93 (0.81-1.06)	0.289
Fibrinolysis	0.52 (0.42-0.64)	<0.001
In-hospital CABG	0.76 (0.63-0.92)	0.004
In-hospital cardiogenic shock	0.72 (0.46-1.10)	0.136
Statin at hospital discharge	3.37 (2.76-4.12)	<0.001
Beta-blocker at hospital discharge	1.48 (1.23-1.78)	<0.001
ACE inhibitor/ARB at hospital discharge	1.55 (1.31-1.83)	<0.001
MI at follow-up	0.66 (0.55-0.79)	<0.001

CI: Confidence interval, PCI: Percutaneous coronary intervention, CABG: Coronary artery bypass grafting, MI: Myocardial infarction, HF: Heart failure, STEMI: ST-elevation myocardial infarction, ACE: Angiotensin-converting enzyme, ARB: Angiotensin-receptor blocker, MI: Acute myocardial infarction. Hazard ratios (95% CI) were obtained from the stratified Cox proportional hazards regression models.

**Table 3:** Variables that correlated significantly and independently with actively working at the last contact by Cox regression model 3.

Variables	Hazard-ratio	95% CI	*p-*value
Age > median	0.76	0.54-0.89	0.001
Male sex	1.52	1.24-1.85	<0.001
Government health insurance	1.36	1.16-1.60	<0.001
History of angina	0.69	0.58-0.82	<0.001
History of MI	0.76	0.63-0.92	0.005
Actively smoking	0.81	0.69-0.96	0.015
STEMI	0.81	0.67-0.97	0.021
Anterior-wall MI	0.75	0.64-0.88	0.001
Non-primary PCI	0.77	0.65-0.91	0.002
Fibrinolysis	0.61	0.47-0.78	<0.001
In-hospital cardiogenic shock	0.60	0.38-0.93	0.023
Statin at hospital discharge	3.01	2.43-3.71	<0.001
Beta-blocker at hospital discharge	1.26	1.04-1.53	0.020
ACE inhibitor/ARB at hospital discharge	1.37	1.14-1.65	0.001
MI at follow-up	0.72	0.58-0.88	0.001

Model 3 included all of the variables listed in Table 1 (baseline+in-hospital+hospital discharge/post-discharge) as independent variables. Legends as in Table 1. CI: Confidence interval, MI: Myocardial infarction, STEMI: ST-elevation myocardial infarction, PCI: Percutaneous coronary intervention, ACE: Angiotensin-converting enzyme, ARB: Angiotensin-receptor blocker. Hazard ratios (95% CI) were obtained from the stratified Cox proportional hazards regression models. *p*-values were obtained from the stratified log-rank tests.

## APPENDIX

**Supplemental Table 1:** Variables that correlated significantly and independently with active working at the last contact using Cox regression model 1.

Variables	Hazard-ratio	95% CI	*p-*value
Government health insurance	1.30	1.13–1.50	<0.001
Previous angina	0.67	0.58–0.79	<0.001
History of MI	0.77	0.66–0.91	0.002
STEMI	0.80	0.70–0.92	0.020
Male sex	1.41	1.18–1.67	<0.001

Model 1 included the baseline variables listed in Table 1 as independent variables. CI: confidence interval; MI: myocardial infarction; STEMI: ST-elevation myocardial infarction.

**Supplemental Table 2:** Variables that correlated significantly and independently with active working at the last contact using Cox regression model 2.

Variables	Hazard-ratio	95% CI	*p*-value
Government health insurance	1.41	1.21–1.64	<0.001
Previous angina	0.65	0.55–0.77	<0.001
History of MI	0.79	0.66–0.95	0.014
Smoking	0.85	0.72–0.99	0.045
STEMI	0.76	0.61–0.94	0.012
Anterior-wall MI	0.79	0.67–0.92	0.003
Male sex	1.55	1.27–1.88	<0.001
Age	0.82	0.71–0.96	0.002
Primary PCI	1.44	1.14–1.83	0.002
Fibrinolysis	0.57	0.43–0.75	<0.001
In-hospital CABG	0.78	0.63–0.97	0.024

Model 2 included the baseline and in-hospital variables listed in Table 1 as independent variables. CI: confidence interval; MI: myocardial infarction; PCI: percutaneous coronary intervention; CABG: coronary artery bypass graft.
